# Navigating Pregnancy: Information Sources and Lifestyle Behavior Choices—A Narrative Review

**DOI:** 10.1155/2024/4040825

**Published:** 2024-09-21

**Authors:** Christina Gjestvang, Lene Annette Hagen Haakstad

**Affiliations:** Department of Sports Medicine Norwegian School of Sports Sciences, Oslo 0806, Norway

## Abstract

**Background:** Accessible health information during pregnancy is important to positively affect maternal and fetal health. However, the quality and accuracy of health information can greatly vary across numerous sources. This narrative review is aimed at summarizing the literature on pregnant individuals' information sources and how these sources influence their habits toward GWG, PA, and nutrition. Such data will highlight preferences and needs, reveal challenges, and identify opportunities for improvement.

**Methods:** We searched PubMed for studies published in the last decade. Out of 299 studies initially identified, 20 (16 quantitative and four qualitative) met the eligibility criteria (investigating information sources and their influence on health habits toward GWG, PA, nutrition, pregnant participants, adequate data reporting, and being available in full text).

**Results:** Primary sources of health information varied. The Internet (26%–97%) and healthcare providers (HCPs) (14%–74%) predominated, followed by family/friends (12%71%), books/magazines (49%–65%), and guidelines/brochures (25%–53%). Despite the widespread use of the Internet, HCPs were considered the most reliable source. The use of the Internet to retrieve health information was reported to be 2–4 h a week, and < 50% discussed the online information with their HCP. The Internet was also used as a supplementary resource on topics raised by HCPs. Regarding the influence on health habits, the Internet, HCPs, media, and family positively influenced GWG and promoted adherence to recommended guidelines (OR = 0.55–15.5). Only one study showed a positive association between Internet use and PA level. The Internet, media, HCPs, and information brochures were associated with better adherence to nutritional recommendations.

**Conclusions:** Pregnant individuals relied on the Internet and HCP, with a preference for the Internet despite trust in midwives. Several sources of health information were positively associated with adherence to GWG and nutrition recommendations. Improving the quality of online information should be a priority for policymakers and health authorities.

## 1. Introduction

Adequate gestational weight gain (GWG), physical activity (PA), and healthy eating during pregnancy offer health benefits for the mother and baby, such as reducing the risk of gestational diabetes [1–3], pregnancy-induced hypertension [1], premature birth [2, 4], macrosomia [2, 5], and small for gestational age infants [1, 2, 4]. Therefore, providing information on these factors during pregnancy is important in antenatal care [6].

Pregnancy increases the risk of weight gain, and optimal GWG may be important to prevent later overweight and obesity in individuals [7]. A GWG greater than or less than the recommended range also increases the risk of maternal and infant complications [8, 9]. Current IOM GWG guidelines recommend a maximum weight gain of 12.5 to 18.0 kg for underweight (BMI: < 18.5), 11.5 to 16.0 kg for normal weight (BMI: 18.5 to 24.9), 7.0 to 11.5 kg for overweight (BMI: 25.0 to 29.9), and 5.0 to 9.0 kg for obese (BMI: ≥ 30) individuals [8]. Approximately 37% to 47% of pregnant individuals exceed these guidelines [9, 10].

PA and healthy eating may help pregnant individuals reach the recommended GWG and reduce pregnancy complications [1, 6, 11]. In a healthy pregnancy, American College of Obstetrics and Gynecology (ACOG) guidelines recommend that pregnant individuals should do at least 150 min of moderate-intensity aerobic PA/week, incorporating both aerobic and muscle-strengthening activities [6]. However, research shows that PA tends to decline from prepregnancy levels and continues to decrease throughout pregnancy, with only a minority of pregnant individuals (approximately 50% to 85%) maintaining regular PA levels throughout the second and third trimesters [10, 12, 13]. Further, during pregnancy, healthy eating, including adequate energy intake and a variety of foods to meet maternal and fetal needs, can lead to fewer pregnancy outcomes and adverse child health outcomes, including positive effects on fetal growth, obstetrical outcomes, and perinatal survival [11]. There is also a significant proportion of food safety advice due to the increased risk of foodborne illness [11]. As such, pregnant individuals often report wanting information about healthy eating to modify their diet to optimize both fetal and maternal outcomes [14].

There are a wide variety of information sources on GWG, PA, and nutrition during pregnancy. However, the quality and accuracy of health information can vary widely, not only between different sources, but also within them. The quality of health information on the Internet is a major concern, especially given the abundance of information and the ease of access [15]. Without regulation, anyone can publish information on the Internet, which means that the information may not be accurate and reliable. To counteract this, healthcare providers (midwives, obstetricians, or general practitioners) may play an essential role in providing pregnant individuals with reliable information about GWG, PA, and nutrition. A recent systematic review showed that pregnant individuals trust and follow the advice given by healthcare providers [16]. However, studies show that healthcare providers lack knowledge about how to educate and support pregnant individuals in making healthy lifestyle choices [17–19]. This is further supported by various studies that highlight the lack of communication between pregnant individuals and their healthcare providers on these topics [20–22].

Due to the varying quality of health information among pregnant individuals, it is essential to investigate the impact of these sources on health habits. Such data will shed light on the preferences, needs, and challenges faced by pregnant individuals, as well as identify opportunities for improvement. Hence, this narrative review is aimed at describing and discussing the literature on sources of information for pregnant individuals and how this influences their health habits toward GWG, PA, and nutrition. To our knowledge, there is no scientific literature that provides such an overview and we aimed to fill this gap by examining and synthesizing existing research in this area.

## 2. Materials and Methods

To address the research question, we searched PubMed on the 1^st^ of August 2023. The search strategy in PubMed included the following terms: (pregnancy OR pregnancy [mesh] or «pregnant women» OR «pregnant women» [mesh]) AND («information sourc∗» OR «sources of information») AND («weight gain∗» OR «weight gain» [mesh] OR «gestational weight gain∗» OR «gestational weight gain∗» [mesh] OR nutrition OR diet∗ OR diet∗ [mesh] OR «physical activit∗» OR exercis∗ OR exercis∗ [mesh] OR training) AND (behaviour∗ OR behavior OR behavior [mesh] OR «information behavior∗» OR «behavior change∗» OR «health behavior∗» OR «health behavior∗» [mesh] OR «feeding behavior∗» OR «feeding behavior∗» [mesh]). The search was narrowed to studies published in the past decade (after 2012).

The inclusion criteria for the reviewed articles were studies investigating sources of information among pregnant individuals or how information sources influenced their habits about GWG, PA, and nutrition. Studies that were not available in full text, did not include pregnant participants, or were written in languages other than English, Norwegian, Swedish, or Danish were excluded.

**Figure 1:**
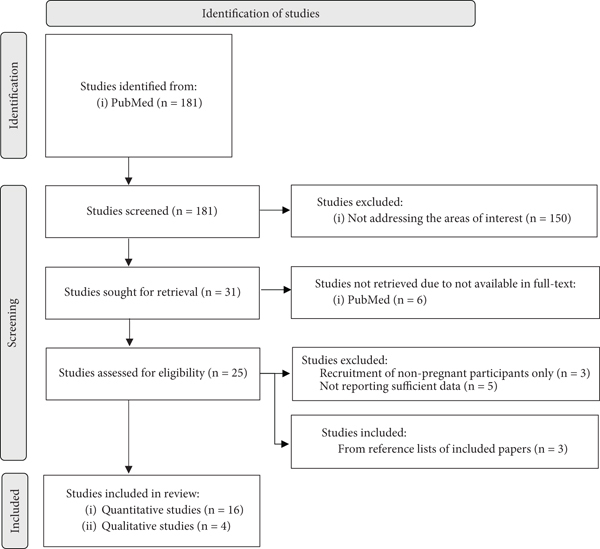
Flow chart of study selection.

Out of 181 studies initially identified, we excluded 150 studies that did not address the areas of interest (e.g., not measuring outcomes such as GWG, PA, and nutrition), six studies that were not available in full text, and three studies recruiting nonpregnant participants only. Additionally, we excluded five studies that did not report sufficient data on information sources, leaving us with 17 studies. We also identified three relevant studies from the reference lists of the included papers, resulting in a total of 20 included studies (16 quantitative and four qualitative). The process of study selection is documented in a flow chart (Figure 1) adopted by PRISMA [23, 24], designed to visually represent how the literature search was done, including the numbers of studies identified, included and excluded studies, and the reasons for exclusions [23]. An overview of the included studies is shown in Table 1.

## 3. Results

### 3.1. Quantitative Studies

#### 3.1.1. Population Characteristics

Quantitative studies consisted of 14 cross-sectional studies [10, 25–37], one follow-up study after a randomized controlled trial [38], and one systematic review [39], including participants from multiple countries: United States [29, 32, 38], Australia [28, 31, 37], United Kingdom [27, 35], Canada [26, 36], Norway [10], New Zealand [25], The Netherlands [30], South Africa [33], and Indonesia [34]. The systematic review included participants from the United Kingdom, China, Sweden, Mexico, Italy, Turkey, and the United States [39]. The quantitative studies used electronic [10, 26, 29–31, 34–37], paper-based [28, 29, 32, 38], or interview-administered questionnaires [32, 33] for the assessment of background variables, information sources, and habits regarding GWG, PA, and nutrition during pregnancy. Two studies did not report the format of the questionnaire used [25, 27]. There was a variation in the gestational week at which participants were recruited or responded to the questionnaire. Four studies recruited pregnant individuals in the first [26, 27, 31, 39], nine studies in the second [10, 26–28, 32, 35, 37–39], and six studies in the third [10, 26, 27, 32, 35, 39] trimesters. The mean gestational week ranged from seven [31] to 39 [26]. Huberty et al. [29] recruited 62% of the participants in the third trimester, without data on the remaining individuals. Five studies did not report the trimester or mean gestational week [25, 30, 33, 34, 36]. Only one study [32] reported recruiting only primiparous individuals, while the proportion of primiparous individuals in the other studies ranged from 41% [27] to 86% (48). Ten studies included a mixed sample: individuals who were pregnant or had given birth within 2 months to 5 years postpartum [25, 26, 28–30, 34–36, 38, 39].

The studies showed diversity in participant demographics, and the mean age ranged from 27.0 ± 6.2 [33] to 34.0 ± 4.2 years [26]. The proportion of individuals holding a university degree varied from 17% [39] to 92% [36], and the employment rates ranged from 13% [28] to 96% [10]. Two studies reported that 49% to 70% of the participants were classified as having low socioeconomic status [31, 32]. Ethnicity percentages also varied across the studies, and the proportion of Caucasian ethnicity ranged from 38% [27] to 99% [37]. One study reported enrolling a majority of participants of African descent (86%) [33]. Seven studies [10, 26, 32, 33, 36–38] provided data on participant's body mass index (BMI, kg/m^2^) with the prevalence of overweight or obese individuals ranging from 13% [33] to 49% [38]. In four included studies reporting PA behavior, 20% [26] to 88% [10] were engaged in PA or exercise before pregnancy [10, 26, 33, 36].

#### 3.1.2. Information Sources

The primary sources of health information varied widely in the quantitative studies. The Internet (26%–97%) [10, 26–32, 34, 35, 37–39] and healthcare providers (14-74%) [10, 25–28, 31, 33–35, 37, 38] predominated, followed by family/friends (12%–71%) [25, 31–35, 37], books/magazines (49%–65%) [33, 34, 37, 38], and guidelines/information brochures (25%–53%) [25, 26]. Pregnant individuals used approximately 2–4 h per week to search for pregnancy-related information on the Internet [26, 30], highlighting the role of the Internet in providing health information [10, 26, 27, 29, 30, 37–39]. Less than half (30% [39] to 50% [30]) discussed the information that they had retrieved from the Internet with their healthcare provider, and the Internet was used as a supplementary resource on topics brought up by healthcare providers [30]. Jacobs, van Steijn, and van Pampus [30] reported that the majority (96%) of pregnant individuals made pregnancy-related decisions based on Internet information.

#### 3.1.3. GWG

Three studies examined how information sources affected GWG. Dalhaug and Haakstad [10] found that reporting the Internet and the media as primary sources of information on GWG was associated with gaining weight below (OR = 15.5) the IOM GWG guidelines, while advice from friends and family was associated with gaining weight above the IOM GWG guidelines. Souza et al. [36] found that advice from family or friends about GWG was linked to gaining both below (OR = 1.5) and above (OR = 1.4) the IOM GWG guidelines. Lastly, Mercado et al. [38] found that information from healthcare providers resulted in a lower likelihood (OR = 0.55) of exceeding the IOM GWG guidelines, independent of prepregnancy weight status.

#### 3.1.4. PA

Two studies examined how information sources influenced PA levels during pregnancy [10, 29]. Huberty et al. [29] found that half of the individuals (48%) decreased their PA level during pregnancy. However, among those who reported the Internet as the main source of information, 26% reported increased PA levels as a result of the information they retrieved, while only 4% reported decreased PA levels [29]. The participants also reported an increase in their level of confidence in PA after using the Internet [29]. In the study by Dalhaug and Haakstad [10], less than 50% reported following the PA recommendations during pregnancy, and no association was observed between the sources of information and the odds of meeting PA recommendations.

#### 3.1.5. Nutrition

Four studies examined how information sources influenced nutrition behavior during pregnancy [10, 25, 27, 29]. Brown et al. [25] found that pregnant participants altered their diets according to the advice of healthcare providers and the recommendations outlined in pregnancy guidelines. The participants reported adding, limiting, and avoiding certain foods, such as alcohol (92%), raw milk products (86%), and raw, smoked, or precooked seafood and fish (84%) [25]. Similarly, Dalhaug and Haakstad [10] found that those who used the Internet and media as primary source of information were more likely to comply with nutritional recommendations, compared with information from family and friends or healthcare providers. Further, Funnell et al. [27] reported that pregnant individuals who used the Internet to retrieve nutrition information reported healthier eating during pregnancy (such as increased fruit and vegetable consumption, folic acid, and vitamin D). Huberty et al. [29] reported that the majority (92%) reported changing their diet during pregnancy and participants reported an increase in their level of confidence in healthy eating after using the Internet.

### 3.2. Qualitative Studies

#### 3.2.1. Population Characteristics

The four qualitative studies used focus groups [40–42] and in-depth interviews [42, 43], with participants from Norway (*n* = 17) [43], the United States (*n* = 82) [41, 42], and France (*n* = 40) [40]. Garnweidner, Pettersen, and Mosdol [43] recruited participants in the second trimester (mean gestational week: 19), Bianchi et al. [40] enrolled participants in all trimesters (second trimester 50%, *n* = 20), and Criss et al. [42] and Criss et al. [41] included pregnant participants in the second trimester (mean gestational week: 23 ± n.r. and 22.2 ± 7.8) or within six to 24 months postpartum. The proportion of participants having their first baby ranged from 50% [40] to 61% [41].

The mean age of the participants was similar to that of the participants in the quantitative studies (26 ± n.r. to 34 ± n.r. years) [41, 42]. The level of education was reported in three studies: high school graduate (69%) [41], college degree or higher level of education (80%) [42], and university degree (59%) [43]. Garnweidner, Pettersen, and Mosdol reported that 71% of the participants were employed. There was variation among the studies regarding ethnicity, with the recruitment of participants classified as Caucasian ethnicity (29%) [43], European descent (66%) [42], African-American (15%) [42], and Hispanic (39%) [41].

#### 3.2.2. Information Sources

Pregnant individuals navigated between different sources of information, and the main sources were the Internet [40, 41, 43], healthcare providers [40–43], friends and family [40–43], and television [40, 41]. In all studies, healthcare providers were reported as the most trusted and reliable source [40–43]. However, the Internet appeared to be the most frequent source of information [42, 43]. According to Garnweidner, Pettersen, and Mosdol [43], pregnant individuals often failed to critically evaluate the quality of information they found on the Internet. Criss et al. [42] found that the participants trusted pregnancy-related websites from national medical organizations but did not trust comment sections on social media (e.g., Facebook). Also, the participants highlighted the importance of validating pregnancy-related information by checking multiple sources for consistency [41].

#### 3.2.3. Nutrition

Only one study investigated whether information sources influenced health behavior among pregnant individuals [40]. This study found that conflicting information from various sources led to confusion and limited the adoption of healthy eating. Participants trusted healthcare providers but were dissatisfied due to few conversations about nutrition. Specifically, they expressed frustration over the lack of clear explanations on which foods to avoid [40]. Although family and friends were not commonly considered reliable sources of information, pregnant individuals tended to be more open to adopting nutrition habits passed down from their mothers [40].

**Figure 2:**
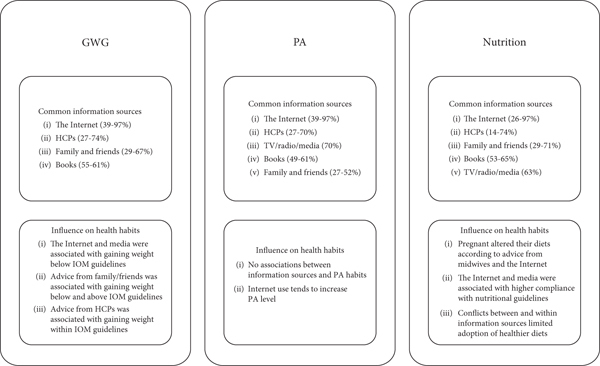
Conceptual model of common information sources and their influence on GWG, PA, and nutrition habits.

A conceptual model of common information sources and their influence on GWG, PA, and nutrition habits is shown in Figure 2.

## 4. Discussion

In this narrative review, we aimed to explore the sources of pregnancy-related health information and their influence on GWG, PA, and nutrition among pregnant individuals. As shown in the conceptual model in Figure 2, our findings revealed that pregnant individuals' primary information sources were the Internet and healthcare providers. Generally, healthcare providers were reported as the most trusted and reliable source, yet, the Internet appeared to be the most frequently used information source. Approximately half of the pregnant individuals discussed the information that they had retrieved on the Internet with their healthcare provider. Our review suggests that the Internet, healthcare providers, media, and family promote adherence to IOM GWG guidelines, while the Internet, media, healthcare providers, and information brochures are associated with better adherence to nutritional recommendations. Only one study showed that the Internet may increase PA levels.

Having a baby is life changing, and pregnancy is a crucial period in which expectant mothers seek information to make informed decisions about their health and the well-being of their unborn child [15, 16]. In the digital age, the Internet has become the primary source of pregnancy-related information, often exceeding healthcare providers due to its convenient accessibility and quick information retrieval [15]. Also, in our review, the Internet was found to be the most common source of information. The availability of digital information has expanded significantly, prompting pregnant individuals to explore diverse channels (such as online forums, social media, and websites) to acquire pregnancy-related information. The participants in the studies of Criss et al. [41] and Bianchi et al. [40] highlighted the importance of validating health information by checking multiple sources for consistency. This is also demonstrated in a systematic review, where pregnant individuals and other patient groups commonly used the Internet to supplement advice from healthcare providers, which empowered them to engage as informed, active participants in their own healthcare [44]. Internet users were more likely to be better prepared for consultations with their healthcare provider and perceived online information to increase their autonomy in health decisions [44]. Further, accessing pregnancy-related health information on the Internet offers a degree of flexibility that appeals to many expectant mothers [45]. Since the Internet has expanded the available health information for patient groups, such as pregnant individuals, acknowledging this influence on GWG, PA, and nutrition habits among pregnant individuals is essential for healthcare providers and policymakers [44, 46]. However, the Internet may also negatively affect pregnancy-related health decisions. For instance, participants in Criss et al. [41] emphasized that if the online information was invalid and inconsistent between sources, it hindered health decision-making and the adoption of a healthy lifestyle. Online information that refuses recommended advice from healthcare providers may therefore adversely impact health habits, and concerns may arise about pregnant individuals who rely solely on health information from the Internet [44, 47]. For instance, many individuals seeking health information on the Internet tend to adopt advice without evaluating the content's quality, which is problematic due to the low-quality and frequently inconsistent information on the Internet [47]. This also aligns with the findings of Garnweidner, Pettersen, and Mosdol [43]. A recent investigation targeting pregnant individuals who sought online information about high-intensity interval training (HIIT) revealed a discrepancy between the online information and evidence-based guidelines for prenatal exercise [48]. The authors also identified information gaps such as safety guidelines for modifying exercises based on prepregnancy PA levels and trimesters [48]. Besides, most of the internet channels did not provide any information on contraindications to exercise during pregnancy [48]. Thus, it is important for healthcare providers to guide pregnant individuals to use the Internet in a way that empowers them to ensure that the information they find is valid and consistent [44].

In this review, we found that pregnant individuals relied primarily on healthcare providers, particularly midwives, as their trusted source of pregnancy-related health information [25, 28, 35, 40–43]. Healthcare providers may play an important role in helping pregnant individuals navigate the overwhelming landscape of available information. One study found that pregnant individuals preferred healthcare providers to recommend high-quality online sources for pregnancy-related information [29], highlighting the potential for healthcare providers to guide pregnant individuals in conducting relevant Internet searches [44]. However, studies show that healthcare providers report a lack of knowledge of current guidelines due to time constraints to keep up with the evolving knowledge [17–19, 21, 22]. Midwives have also expressed limited skills in how to communicate with pregnant individuals about GWG, PA, and healthy eating, possibly resulting in missed opportunities to provide valuable advice [17–19]. In addition, pregnant individuals who report receiving advice about PA have stated that the information is confusing, conservative, and not consistent with current PA recommendations [18, 19, 49, 50]. This may be one explanation for why pregnant individuals have reported verifying advice received from healthcare providers through the Internet [29, 30, 40–42]. This perspective is also echoed by midwives, who have raised concerns about the trust pregnant individuals place in online information, possibly influencing the traditional midwife–pregnant relationship [21]. Further, pregnant individuals often face several barriers in their communication with healthcare providers. A recent systematic review [16] found that barriers to seeking information from healthcare providers included feelings of embarrassment discussing sensitive topics (personal experiences or intimate concerns), as well as clinic waiting times [16]. Pregnancy involves major changes in the body, and topics such as weight gain, PA, and nutrition may be sensitive. Thus, the pregnant individual may feel uncomfortable discussing these important issues. Also, healthcare providers frequently miss opportunities to offer advice, which is often attributed to factors such as time restrictions with multiple patients to meet in a short period of time, with limited time for each patient. This may result in limited time for addressing all of the patient's concerns. Pregnant individuals may have many questions and concerns, which may not be fully addressed due to time constraints. For instance, one study found that only 18% of all clinic visits included counseling about PA and nutrition [51]. Also, research indicates that counseling rates for weight management, PA, and nutrition vary by BMI and pregnancy status, as well as provider specialty [50–52]. Pregnant overweight or obese individuals are shown to be 30% less likely to receive counseling compared with nonpregnant overweight or obese individuals [51]. This could potentially contribute to health disparities, as these individuals might be at higher risk of complications related to excessive GWG, low levels of PA, and poor nutrition.

We found that three studies reported different sources of GWG information [10, 36, 38]. Advice from the Internet, healthcare providers, media, and the family positively influenced GWG and promoted adherence to IOM GWG guidelines [10, 36, 38]. The slight differences across studies may be explained by differences in participant demographics and cultural context. For instance, Dalhaug and Haakstad's [10] study was conducted in Norway with a relatively high socioeconomic status in the population [53]. On the other hand, Souza et al. [36] and Mercado et al. [38] recruited Canadian and American participants in North American contexts, with greater socioeconomic status and cultural diversity compared with Norway. Demographics and cultural context may influence the choice of preferred information sources and the impact of advice from the various sources on health habits, uniquely tailored to the specific society [54]. Thus, considering cultural contexts in future research is needed to ensure that the findings are representative of diverse populations. Another explanation for the inconsistent results between these studies may be due to the different study design. Mercado et al. [38] conducted a randomized controlled trial, while Dalhaug and Haakstad [10] and Souza et al. [36] conducted cross-sectional studies. Cross-sectional studies have limitations in drawing precise conclusions, and causality cannot be established [55]. However, we believe that these studies do not contradict each other, since they provide a more comprehensive understanding of how different information sources can influence GWG.

One study in this review found that online information positively influenced PA levels during pregnancy [29]. However, the overall proportion of individuals who decreased their PA level during pregnancy (48%) was still high [29]. Dalhaug and Haakstad [10] also found that PA levels decreased during pregnancy but did not find any association between online information and PA habits. One explanation for the discrepancy between these two studies may be differences in the study aims. Dalhaug and Haakstad [10] aimed to investigate the main sources of information among pregnant individuals, expanding the scope of the study to include several sources, while Huberty et al. [29] examined how pregnant individuals used the Internet for health information and its influence on health habits. Therefore, selection bias may be present [56]. For instance, it may be possible that participants in the study by Huberty et al. [29] already had a particular interest in using the Internet for pregnancy-related PA information, potentially biasing the results in favor of a positive association with PA level. Further, these two studies used different questionnaires, and thereby, the results may be difficult to compare. Low agreement is shown between various questionnaires used to assess PA [57]. Thus, it is still unclear how online information affects the participation of pregnant individuals in PA. Lack of knowledge about PA benefits, concerns about negative health effects on the fetus, and fear of miscarriage may explain why many pregnant individuals consider PA less important during pregnancy [58, 59]. Therefore, considering these concerns about PA, we still believe that targeted online information to increase knowledge about PA could potentially improve PA among pregnant individuals [29, 58, 60]. One systematic review has suggested the potential of online forums and community support networks to encourage PA among pregnant individuals by socializing and sharing experiences with other pregnant individuals and healthcare providers [60]. Further, in the study by Huberty et al. [29], discussions about online PA information with healthcare providers and significant others were found to lead the pregnant individuals to feel more informed and supported in health decision-making [59].

Based on our results, pregnant individuals reported interest in modifying their diets based on various sources, including the Internet, healthcare providers, and information brochures [10, 25, 27]. Also, two studies in our review showed that the use of the Internet and media as an information source was associated with healthier eating during pregnancy [10, 27]. On the other hand, the Internet might provide information that contradicts national guidelines and healthcare provider's advice, for example, the promotion of unrecommended supplements. As shown in two qualitative studies in this review, conflicting information may lead to confusion and adoption of mistaken beliefs and limit an optimal diet during pregnancy [40, 41]. Additionally, pregnant individuals often find themselves in a dilemma in the decision-making process and experience increased anxiety since they do not trust online information [39]. Since we found that only half of pregnant individuals discussed the retrieved online information with their healthcare provider, they may not be aware of mistaken beliefs about pregnancy, reported on the Internet [39]. Hence, it is important with increased of why and how pregnant individuals use the Internet to make decisions about GWG, PA, and nutrition, to aid healthcare providers in supporting pregnant individuals in decision-making by offering help in interpreting and applying the online information [60, 61].

The diversity in demographics and ethnicity of the participants could impact the generalizability of the findings in our systematic review, highlighting the complexity of health habits during pregnancy [62]. Socioeconomic inequalities affect maternal and fetal health, with lower social classes associated with an increased risk of adverse pregnancy outcomes [63]. Additionally, differences in age and education levels may influence access to and interpretation of health information. To decrease pregnancy-related health inequalities, it is crucial to address these factors in the development of pregnancy-related health information [63]. Targeted health information among all pregnant individuals, regardless of demographic background, can ensure that the information is accessible and relevant to all segments of the pregnant population [58].

### 4.1. Strengths and Limitations

The strengths of this narrative review include the synthesis of research from 15 countries and five different continents. Additionally, the inclusion of both quantitative and qualitative studies offers a more diverse perspective and a wider range of research questions, identifying gaps for future studies. This narrative review also provides greater insight into information sources among pregnant individuals and how this influences their health habits toward GWG, PA, and nutrition. This is important for the development of interventions and healthcare strategies that support pregnant individuals to obtain information tailored to their specific needs and preferences. A limitation is that ten of 20 studies included both pregnant and postpartum participants, possibly having different needs and preferences for health information. Further, the studies in this review addressed various aspects of information sources and health habits during pregnancy, including GWG, PA, dietary choices, emotional wellness, Internet use, and healthcare decision-making, which could lower the precision of this review. Lastly, the diversity in participant demographics and ethnicity could potentially impact the generalizability of the findings from this review. However, a key aspect is that the included studies overall consistently reported similar findings regarding most common information sources. Therefore, we believe that the external validity of this review remains robust.

### 4.2. Practical Implications and Future Research

The widespread use of the Internet highlights the need for research evaluating the quality and accuracy of online health information targeted at pregnant individuals and their impact on health habits. Such knowledge may aid in developing quality standards and monitoring systems, enabling pregnant individuals to identify reliable sources that positively influence their health habits. There is also a need for longitudinal studies investigating the effects of digital interventions designed to provide credible health information during pregnancy.

The use of the Internet appears to enhance traditional sources of health information, such as healthcare providers, rather than replace them [44]. Its impact on health decision-making may depend on whether the pregnant individual shares the online information with their healthcare provider and their response to it [44, 46]. Therefore, healthcare providers should acknowledge and encourage open dialogues about the use of the Internet, provide support to clarify concerns retrieved from online information, and improve their ability to communicate regarding GWG, PA, and nutrition. Considering that pregnant individuals often verify advice from healthcare providers on the Internet, research should evaluate the effect of interventions aiming to improve healthcare providers' communication abilities and support.

Research is needed to identify the most effective strategies for overcoming barriers that pregnant individuals face when meeting healthcare providers. Future studies should consider the use of technology and investigate how reliable online health platforms, mobile apps, and information brochures may enhance the traditional midwife–pregnant relationship and provide pregnant individuals with additional support and resources to supplement the advice received during clinical visits. Such interventions could provide customized advice, track progress, and offer support, potentially improving the effectiveness of counseling and overcoming some of the identified barriers.

Lastly, given the diverse socioeconomic status and cultural contexts among pregnant individuals, research examining how these factors influence their information-seeking behaviors is crucial. This will ensure that interventions and strategies are tailored to the needs and contexts of all pregnant individuals, promoting better health outcomes.

## 5. Conclusions

Our narrative review showed that pregnant individuals relied on the Internet and healthcare providers, with a preference for the Internet despite trust in midwives. Less than half discussed the information that they had retrieved on the Internet with their healthcare provider. The Internet, healthcare providers, media, and family positively influenced adherence to IOM GWG guidelines. Regarding healthy eating, the Internet, media, healthcare providers, and information brochures were associated with better adherence to nutritional recommendations. Our review highlights the Internet as an important source of health information during pregnancy. Improving the quality of online information, such as establishing quality standards and monitoring systems for online health information, should be a priority for policymakers and health authorities.

## Figures and Tables

**Table 1:** Overview of included studies.

**Quantitative studies**				
**Authors (year), study design**	**Aims related to the principal objectives of this narrative review**	**Method**	**Participants**	**Main results related to the principal objectives of this narrative review**
Brown et al. (2020), cross-sectional.	Determine individual's dietary choices, food safety practices, and sources of nutrition Information.	• Tool: Questionnaire • Location: New Zealand • Recruitment: Via social media, professional associations, posters, word of mouth, and personal contact.	Pregnant or within 6 months postpartum and breastfeeding (*n* = 458). Characteristics: 32.5 ± 6 years, white (95%), first pregnancy (43%), university degree (83%), and reporting good health (94%).	The most common information sources during pregnancy were midwives (37%) and the New Zealand pregnancy and breastfeeding guidelines (25%). Pregnant made dietary changes due to food safety concerns, such as avoiding alcohol (92%), raw milk products (86%), and raw/smoked/precooked seafood/fish (84%). Dietary changes were often made in response to infant symptoms without supporting evidence.
Da Costa et al. (2015), cross-sectional.	Determine individual's informational needs and preferred sources related to PA and nutrition behaviors.	• Tool: Electronic questionnaire • Location: Canada. • Recruitment: Via research staff or through study fliers in the waiting rooms at the offices of obstetricians/gynecologists affiliated with the McGill University Health Centre (MUHC), the Jewish General Hospital, and St. Mary's Hospital in the Montreal area, as well as local prenatal classes.	Pregnant (13–39-week gestation) or within 2 months postpartum (*n* = 74). Characteristics: 34.0 ± 4.2 years, white (68%), university degree (78%), employed (56%), physically active (20%), and BMI ≥ 25 (32%).	Preferred information sources were the Internet (73%), HCP (62%), and a booklet (53%). The participants reported spending 3.9 h/week on the Internet, with 13.6% rating the information as not at all helpful, 39.2% reporting it as somewhat helpful, and 47.3% indicating that the information was very helpful.
Dalhaug & Haakstad (2019), cross-sectional.	Investigate the main information sources concerning PA, GWG, and nutrition, and evaluate how these information sources affect their health behaviors.	• Tool: Electronic questionnaire. • Location: Norway. • Recruitment: Via social media and 18 antenatal clinics in Oslo, Norway.	Pregnant (mean gestation week: 30.6 ± 5.9) (*n* = 150). Characteristics: 31.1 ± 4.3 years, university degree (64%), employed (96%), physically active before pregnancy (88%), physically active during pregnancy (49%), and BMI ≥ 25 (30%).	The most common information sources for GWG, PA, and nutrition combined were blogs/online forums (39%) and HCP (27%). Most women (54% to 68%) reported the Internet and media sources than HCP (19% to 28%) as the information source with the most impact on their health behavior. Using the Internet and media as primary information sources on GWG increased the odds of gaining weight below the IOM guidelines. Receiving advice from friends/family on GWG was associated with gaining weight above the IOM guidelines. No significant associations were observed between the three groups of information sources and the odds of meeting the PA guidelines. Higher compliance with nutritional guidelines was seen among those citing the internet and media as main information sources.
Funnell et al. (2018), cross-sectional.	Investigate individual's behavior and information sources about nutrition and vitamin supplementation.	• Tool: Questionnaire. • Location: United Kingdom. • Recruitment: Via the antenatal clinic at Croydon University Hospital, antenatal clinic, London, UK.	Pregnant (13–28-week gestation (59%)) (*n* = 133). Characteristics: < 20 to 30 years (49%), first pregnancy (41%), white (38%), university degree (32%), and reporting good health (77%).	The Internet (26%) and HCP (14%) were the most popular information sources. However, the participants preferred to receive information from the antenatal clinic (62%), the Internet (46%), and mobile applications (27%).
Grimes et al. (2014), cross-sectional.	Explore sources of information and identify which sources were used most frequently, considered most useful, and preferred.	• Tool: Paper-based questionnaire. • Location: Australia. • Recruitment: Via Royal Women's Hospital, Melbourne, Australia.	Within 4 months postpartum (*n* = 350). Characteristics: 32.4 ± 4.9 years, mean age of infants: 20.3 ± 3.5 weeks, first pregnancy (62%), university degree (74%), and employed (13%).	Midwives were the most common information source (70%), and 44% used the Internet to access information. Pregnants who received most of their pregnancy care from a midwife described midwives as their most useful source of information (28%). In contrast, of those receiving most of their care from a general practitioner in an antenatal clinic, the largest proportion reported that the Internet was their most useful source of information (28%).
Huberty et al. (2013), cross-sectional.	Determine the use of the Internet for health, PA, and nutrition information.	• Tool: Electronic and paper-based questionnaires. • Location: United States. • Recruitment: Via handouts provided in person at the Women Infant and Children clinics, family physicians, hospital prenatal courses, and websites targeted at pregnant women or moms, and daycares.	Pregnant (42%) or within 12 months postpartum (58%) (*n* = 293). Characteristics: 28.5 ± 4.9 years, university degree (51%), third trimester (62%), and healthy pregnancy (62%).	Almost all (94%) used the Internet as an information source. The participants reported using the Internet six to ten times for general pregnancy-related health information. Half used the internet as an information source related to PA, and some (26%) increased their PA as a result. Findings related to nutrition were similar to PA.
Jacobs et al. (2019), cross-sectional.	Identify how much time individuals search the Internet for information, what information they seek, how much they value this information, and if they discuss the information with healthcare providers.	• Tool: Electronic questionnaire. • Location: Netherlands. • Recruitment: Via social media.	Attempting pregnancy, pregnant, or within 2 months postpartum (*n* = 365). Characteristics: 18 to 30 years (56%), university degree (20%), employed (87%), white (94%), and healthy pregnancy (43%).	The majority of participants (96%) used the Internet as an information source, and most pregnant (70%) reported using the Internet ≤ 2 h/week. Individuals searched the Internet more often in the first (34.5%) and third trimesters (46.4%) and used it mainly because it is easily accessible, and quick for gaining additional information. Over 90% of the participants thought that information from the Internet was reliable, yet, only 50% discussed this information with HCP. Almost half (47%) took the information found on the Internet into account when making decisions regarding their pregnancy, but most relied on information provided by HCP (91.2%) and family/friends (54%).
Lang et al. (2023), cross-sectional.	Explore preferred sources of health information.	• Tool: Electronic questionnaire. • Location: Australia. • Recruitment: Via the Monash Health maternity clinic and Medibank Private Limited.	Pregnant (mean gestation week: 7.0 (*n* = 261)). Characteristics: 30.15 ± 4.72 years, employed (77%), university degree (84%), and low socioeconomic status (70%).	Many reported retrieving health information concerning healthy body weight (57%) and nutrition (eating a healthy diet (62%), folic acid (62%), and prepregnancy multivitamins (59%)). Preferred information sources included HCP (74%) and the Internet (66%), followed by family/friends (29%).
Lindsay et al. (2021), cross-sectional.	Assess sources of information about GWG, nutrition, and PA.	• Tool: Questionnaire completed in-person or via telephone. • Location: United States • Recruitment: Via research staff, Brazilian immigrants, and social media.	Pregnant (mean gestation week: 27.5 ± 5.6) (*n* = 86)). Characteristics: 28.3 ± 4.7 years, first pregnancy (100%), born in Brazil (97%), living in the United States for an average of 10.7 ± 7.3, low socioeconomic status (49%), and BMI ≥ 25 (26%).	Many reported actively seeking information from the Internet (GWG (72%), nutrition (81%), and PA (76%)) and from family/friends (GWG (67%), nutrition (71%), and PA (52%)).
Mercado et al. (2017), follow-up after a randomized controlled trial.	Investigate sources of information about nutrition, PA, and weight and the impact of information sources on maternal GWG.	• Tool: Paper-based questionnaire. • Location: United States. • Recruitment: Via research assistants or healthcare providers during the first prenatal visit at six obstetric offices in Rhode Island.	Within 6 weeks postpartum (mean gestation week at enrollment: 13.5 ± 1.8 (*n* = 401)). Characteristics: 28.8 ± 5.2 years, white (83%), university degree (60%), and BMI ≥ 25 (49%).	Most pregnant reported receiving information from a book (61%) or the Internet (58%), followed by general practitioners (56%), dietitians (48%), and nurses (34%). Only information from general practitioners was associated with reduced odds of excessive GWG.
Okafor & Goon (2021), cross-sectional.	Examine the beliefs about and perceived benefits of PA during pregnancy, and to identify sources of information on PA.	• Tool: Interview-administered questionnaire. • Location: South Africa. • Recruitment: Via healthcare clinics offering antenatal health services in Buffalo City, Eastern Cape province.	Pregnant (*n* = 1082). Characteristics: 27.0 ± 6.2 years, black (86%), university degree (18%), employed (32%), reporting good health (59%), BMI ≥ 25 (13%), first pregnancy (30%), and physically active (35%).	The most common information sources on PA were television, radio/other media (70%), followed by books/newspapers/magazines (49%), HCP (47%), family (45%), friends (27%), and other women (18%).
Rahmawati et al. (2021), cross-sectional.	Investigate the experience of seeking and obtaining nutritional information and its relationship to individual's sociodemographic and pregnancy characteristics.	• Tool: Electronic questionnaire. • Location: Indonesia. • Recruitment: Via the first author's social media sites and public social media groups in Malang City, Indonesia.	Within 24 months postpartum (*n* = 335). Characteristics: 30.0 ± 4.8 years, first pregnancy (47%), child's age: 10.3 ± 6.5 months, university degree (59%), and employed (44%).	With a frequency of receiving information > 10 times during pregnancy, the most common information sources were husbands (70%), the Internet (60%), and social media (55%). With a frequency of receiving information 1–10 times during pregnancy, books (65%), obstetricians (64%), family (64%), and TV/radio (63%) were the most common sources. Information from friends (33%), books (30%) obstetricians (25%), family (25%), and midwives (10%) were more rarely. The most trusted sources of information were obstetricians (62%), followed by midwives (12%) and nutritionists (12%).
Sayakhot & Carolan-Olah (2016), systematic review.	Describe access and use of the Internet as the source of information.	• Tool: Systematic literature review. • Locations: United Kingdom, China, Sweden, Mexico, Italy, Turkey, and United States. • Recruitment: Via web-based questionnaires (two studies) and from waiting rooms of outpatient antenatal clinics (five studies).	Currently pregnant (42% to 62%) or up to 1 year postpartum ((38% to 58%) *n* = 3359 from seven different studies (*n* = 182 to 1347 in each study)). Characteristics: 17–49 years, university degree (17% to 80%), employed (22% to 77%), reporting good health (62% to 80%), first pregnancy (47% to 86%), first trimester (21%), second trimester 47%), and third trimester (32% to 100%).	The Internet was the most common (44% to 97%) information source and was perceived as useful and reliable. The information was related to various topics related to pregnancy, including stages of childbirth, fetal development, and nutrition. Few participants discussed information found on the Internet with HCP.
Soltani et al. (2017), cross-sectional.	Explore nutrition practices and information sources.	• Tool: Electronic questionnaire. • Location: United Kingdom. • Recruitment: Via Tommy's (the UK-based baby charity) through networking systems with a wide range of organizations and support groups.	Currently pregnant (28%, *n* = 58, mean gestation week: 25.6 ± 10.2) or previously given birth (72%, *n* = 147) (*n* = 205). Characteristics: > 20 years (74%), white (95%), first pregnancy (60%).	The main information sources were HCP (68%), bounty pack (50%), family (39%), and the Internet (37%). When ranking their favorite sources of information, HCP was the most popular (38%). Smartphone applications (apps) and recipe booklets were suggested by over 50% of participants as a new addition to existing services.
Souza et al. (2020), cross-sectional.	Identify potential changes in nutrition behaviors that may be contributing to suboptimal GWG.	• Tool: Electronic questionnaire. • Location: Canada. • Recruitment: Via snowball sampling through social media with secure links to the survey.	Currently pregnant (19%, *n* = 220) or within 5 years postpartum (81%, *n* = 951) (*n* = 1171). Characteristics: 18 to 30 years (90%), university degree (92%), employed (89%), physically active (57%), and BMI ≥ 25 (31%).	Receiving advice from family/friends about GWG was associated with gaining below (OR = 1.5) and above (OR = 1.4) IOM guidelines when compared to pregnant who did not receive advice from family/friends.
Willcox et al. (2015), cross-sectional.	Investigate GWG information sources and how they varied by key demographic factors.	• Tool: Electronic questionnaire. • Location: Australia. • Recruitment: Via emails carried out at a major Australian maternity tertiary training hospital.	Pregnant (mean gestation week: 20.8 ± 5.5, *n* = 368). Characteristics: 32.5 ± 4.5 years, white (99%), first pregnancy (47%), university degree (62%), employed (69%), and BMI ≥ 25 (36%).	More than half had sought for GWG information (55%). Most frequently reported sources were the Internet (83%), followed by books (55%), friends (52%), general practitioners (45%), and family (43%). The Internet was also ranked as the most important source of information (33%), followed by general practitioners (17%), books (15%), and obstetricians (12%). Only 10% recalled receiving GWG guidelines from HCP, and 54% were consistent with IOM guidelines.

Qualitative studies				
Authors (year), study design	Aims	Method	Participants	Main results
Bianchi et al. (2016), qualitative.	To better understand the determinants of eating behaviors by focusing on pregnant nutrition-related information-seeking practices.	• Tool: Focus groups (*n* = 7). • Location: France. • Recruitment: Not reported, but participants were recruited in Paris and Provence, France.	Pregnant (second trimester 50%, *n* = 20, *n* = 40). Characteristics: 30.5 ± 4.2 years, first pregnancy (50%), mean BMI: 22.2 ± 3.4, and not developed gestational diabetes.	The most common nutrition-related information sources were HCPs, the social environment (e.g., family), and the mass media (e.g., TV or the Internet). The participants strongly trusted their HCP who appeared to be the gold standard. The social environment and mass media were not perceived as a reliable source of information. However, HCP did not spend much time discussing nutrition-related issues and quickly referred to dietary restrictions, being focused mainly on GWG. Participants tried to deal with the information that they obtained from all these sources, and conflicts between and within information sources resulted in confusion that limited the adoption of healthier eating behavior.
Criss et al. (2015), qualitative.	Explore how health information sources inform decision-making.	• Tool: Focus groups (*n* = 7). • Location: United States. • Recruitment: The Eastern Massachusetts community health center and by telephone.	Pregnant (mean gestation week: 22.2 ± 7.8, 35%, *n* = 17) or within 24 months postpartum (65%, *n* = 32 (*n* = 49)). Characteristics: 26.4 ± 6.6 years, first child (61%), high school graduate (69%), and US-born (39%).	Common health information sources included HCP, family, the Internet (Google and ), social media (YouTube and Facebook), and TV. Many cited their HCP as their trusted information source. Further, most participants reported that family was a vital source of information and stated that they trust their mothers, along with other female family members including grandmothers, sisters, aunts, and mother-in-laws. They also highlighted the importance of validating health information by checking multiple sources for consistency and resolving contradictory information.
Criss et al. (2016), qualitative.	Explore the perceptions of factors that affect GWG, including nutrition, PA, and information sources and content, and the experiences with HCP and received advice regarding GWG.	• Tool: Focus groups (*n* = 7) and in-depth interviews (*n* = 9). • Location: United States. • Recruitment: Via Atrius Harvard Vanguard Medical Associates, a large multicenter clinical practice in Boston Area, USA.	Pregnant (mean gestation week: 23, *n* = 33). Characteristics: 34 years, European descent (66%), African-American (15%), college degree or higher level of education (80%), and BMI ≥ 25 (52%).	The main information sources were HCP, family, and friends. Most participants reported that HCPs were the most trusted and reliable source. Pregnants highly valued discussions with their HCP to set GWG goals. Several participants also reported reading pregnancy websites, apps, and weekly emails. The individuals trusted pregnancy-related websites from national medical organizations, but most did not trust comment sections on such websites or Facebook.
Garnweidner et al. (2013), qualitative.	Explore experiences with nutrition-related information.	• Tool: In-depth interviews (*n* = 17). • Location: Norway. • Recruitment: Via midwives during antenatal care at eight different Mother and Child Health Centers in Oslo Area, Norway.	Pregnant (mean gestation week: 19, *n* = 17). Characteristics: 28 years, white (29%), university degree (59%), and employed (71%).	The participants had actively sought information during their pregnancy, especially in the first trimester. Pregnant navigated between different sources of information: from the Internet, midwives, friends, colleagues, or family. The internet appeared to be the most frequent source of information, and the participants did not seem to critically evaluate the quality of the information found. Very few participants stated that it was important to use the information found on websites from official health institutions. Midwives were reported as the most reliable and trustworthy source of information. Information from family was especially common among participants with immigrant backgrounds, also experiencing contradictory advice as challenging.

Abbreviations: GWG = gestational weight gain, HCP = healthcare providers, PA = physical activity.

## Data Availability

Data sharing is not applicable to this article as no datasets were generated or analysed.
