# Supplementary material for: Navigating Pregnancy: Information Sources and Lifestyle Behavior Choices—A Narrative Review
Source: J Pregnancy. 2024 Sep 21;2024:4040825. doi: 10.1155/2024/4040825 (PMC11438513; doi:10.1155/2024/4040825)
Supplement: Supporting Information — Additional supporting information can be found online in the Supporting Information section. Narrative review checklist. [file 4040825.f1.pdf]

Academy of Nutrition and Dietetics

Narrative Review Checklist

Section/topic	#	Checklist item	Reported on page or line #
**TITLE**			pp. 1 & 3
Title	1	Identify the report as a Narrative Review of …	
**ABSTRACT**			
Unstructured summary	2	Provide an unstructured summary including, as applicable: background, objective, brief summary of narrative review and implications for future research, and clinical practice or policy development.	pp. 1
**INTRODUCTION**			
Rationale/background	3	Describe the rationale for the review in the context of what is already known.	pp. 2-3
Objectives	4	Specify the key question(s) identified for the review topic.	pp. 3
**METHODS**			
Research selection	5	Specify the process for identifying the literature search (eg, years considered, language, publication status, study design, and databases of coverage).	pp. 4
**DISCUSSION/SUMMARY**			
Narrative	6	Discuss: 1) research reviewed including fundamental or key findings, 2) limitations and/or quality of research reviewed, and 3) need for future research.	pp. 5-10
Summary	7	Provide an overall interpretation of the narrative review in the context of clinical practice and/or the Nutrition Care Process for registered dietitian nutritionists, clinical practice for other health professionals, policy development and implementation, or future research.	pp. 5-10
